# Prevalence and Clinical Picture of Sleep Paralysis in a Polish Student Sample

**DOI:** 10.3390/ijerph17103529

**Published:** 2020-05-18

**Authors:** Paulina Wróbel-Knybel, Hanna Karakuła-Juchnowicz, Michał Flis, Joanna Rog, Devon E. Hinton, Piotr Boguta, Baland Jalal

**Affiliations:** 1 Ist Department of Psychiatry, Psychotherapy and Early Intervention, Medical University of Lublin, 20-059 Lublin, Poland; karakula.hanna@gmail.com (H.K.-J.); drmichael@wp.pl (M.F.); rog.joann@gmail.com (J.R.); 2 Department of Clinical Neuropsychiatry, Medical University of Lublin, 20-059 Lublin, Poland; 3 Massachusetts General Hospital, 55 Fruit St, Boston, MA 02114, USA; devon_hinton@hms.harvard.edu; 4 Department of Psychiatry, Harvard Medical School, Boston, MA 02115, USA; 5 Locum Pharmacy for Well and Lloyds in Berkshire, Devon, Dorset, Hampshire, Oxfordshire, West Sussex, Wilthshire RG30 2BT, UK; piotr.boguta@hotmail.com; 6 Behavioural and Clinical Neuroscience Institute and Department of Psychiatry, University of Cambridge, Cambridge CB2 0QQ, UK; bj272@cam.ac.uk

**Keywords:** sleep paralysis, psychopathologic symptoms, anxiety, parasomnia, sleep disorder, PTSD6, fear

## Abstract

Sleep paralysis (SP) is a psychobiological phenomenon caused by temporary desynchrony in the architecture of rapid eye movement (REM) sleep. It affects approximately 7.6% of the general population during their lifetime. The aim of this study was to assess (1) the prevalence of SP among Polish students in Lublin (*n* = 439) using self-reported online surveys, (2) the frequency of SP-related somatic and psychopathologic symptoms, and (3) the factors potentially affecting the occurrence of symptoms among people experiencing SP. We found that the incidence of SP in the Polish student population was slightly higher (32%) than the average prevalence found in other student populations (28.3%). The SP clinical picture was dominated by somatic symptomatology: 94% of respondents reported somatic symptoms (most commonly tachycardia, 76%), 93% reported fear (most commonly fear of death, 46%), and 66% reported hallucinations (most commonly visual hallucinations, 37%). The number of SP episodes was related to sleep duration and supine position during sleep. The severity of somatic symptoms correlated with lifestyle variables and anxiety symptomatology. Our study shows that a significant proportion of students experience recurrent SP and that this phenomenon is associated with fear and physical discomfort. The scale of the phenomenon requires a deeper analysis.

## 1. Introduction

Sleep paralysis (SP) is a common condition that affects approximately 7.6% of the general population during their lifetime [1]. The episodes happen either during sleep onset or offset and entail gross motor paralysis coupled with full consciousness of the situation. Although the pathophysiology of SP is not well understood, the occurrence of SP is related to a desynchrony in the architecture of rapid eye movement (REM) sleep [2,3,4]. In polysomnography, this state is marked by plentiful alpha EEG or a mixed pattern of alpha waves and low voltage slow waves and persistence of muscle atonia [5]. Body paralysis during SP is triggered by the pons and ventromedial medulla that inhibit the motor neurons during REM sleep by γ-aminobutyric acid and glycine [2].

SP is usually accompanied by somatic sensations, such as chest pain, shortness of breath, heart palpitations, feeling of choking, sweating, trembling, light-headedness, and nausea. These experiences are variously classified and labelled by researchers, for example, panic symptoms [6], somatic symptoms [7], or vegetative symptoms [8]. 

SP is often accompanied by visual, tactile, as well as auditory hallucinations [9,10]. SP hallucinations are commonly classified into three types: (1) the “intruder” associated with a sense of a menacing presence, (2) the “incubus” associated with a feeling of tightness on the chest, and (3) unusual bodily sensations such as feelings of levitation and other types of OBEs (out-of-body experiences) [9]. 

SP hallucinations have generated different interpretations that vary depending on the culture in which they occur. In China, SP is understood as “ghost oppression” [11]. In Japan, it is thought to be caused by one of the Buddhist deities referred to as Fudoh-Myohoh (“Kanashibari”) [12]. In the United States, it is sometimes interpreted as alien abduction [13]. In Egypt, it is described as an attack by the evil genies (i.e., the jinn) [14]; in Turkey, as a Karabasan attack (spirit like creatures) [15]; in Newfoundland, as an old witch (“old hag phenomenon”) [16]; in Italy, as a pandafeche attack (witches and cat-like creatures) [17]; in South Africa, as an attack by the Thokoloshe [18]; in Brazil, as a crone with long fingernails who tramples on the chest (“Pisadeira”) [19]; and in Cambodia, as an attack by a ghost, with SP called “the ghost pushes you down” [6]. These cultural explanations of SP may affect the rate and severity of SP episodes.

SP is often associated with severe anxiety [1,9,20,21,22], and cultural interpretations of SP (e.g., as a supernatural experience) seem to exacerbate its severity. In Egypt, where SP is regarded as a supernatural experience, 50% of respondents reported fear of death. However, in Demark, where SP is interpreted as a psychological phenomenon, only 17% reported such fears [23]. Thus, the geographic and cultural bases of SP can have differing affects across different cultures.

SP is more common in people with narcolepsy and other psychiatric disorders, especially in those with anxiety disorders such as panic disorders (between 20.8% to 30.6%) [24], social phobias (22.2%) [24], or generalized anxiety disorders (15.8%) [24]. Some studies show an increased incidence of SP in people with post-traumatic stress disorder (PTSD) [14,25], 67% in a Cambodian sample [6]. Research also indicates that depression is strongly associated with SP in a multiple predictor model [26] and that people who experience SP have higher symptoms of depression compared to people who have never experienced SP [27,28]. Sharpless et al., 2010, find a significant correlation between depression symptoms and recurrent fearful SP [29]. Body position during sleep onset seems relevant as SP is more common when sleeping in a supine position [9]. Moreover, SP prevalence rates are significantly higher in the student population (28.3%) compared to the general population (7.6%) [1]. SP that occurs independently of sleep disorders is referred to as isolated sleep paralysis [30]. The International Classification of Sleep Disorders (ICSD) classification distinguished Recurrent Isolated Sleep Paralysis (RISP) from regular sleep paralysis, placing it in the group of parasomnias related to the REM sleep phase [31]. Moreover, in the latest version of the International Classification of Diseases (ICD-11), RISP was defined and classified under the code 7B01.1 [32]. In the fifth edition of Diagnostic and Statistical Manual of Mental Disorders (DSM-5), RISP is not included, although it can be marked 307.40 (unspecified sleep-wake disorder) or 307.49 (other specified sleep-wake disorders) [33]. 

To date, no published studies have examined SP in Poland. The aim of the current study is to assess (1) the prevalence of SP in a Polish student population, (2) the occurrence of SP-related somatic and clinical symptoms, and (3) the factors contributing to the occurrence of somatic and clinical symptoms among SP experiencers.

## 2. Materials and Methods 

### 2.1. Participants

The study was designed as a cross-sectional questionnaire-based descriptive study. The questionnaire was sent via Facebook to 2500 randomly selected students from various universities (and different years of study) in the Lublin Province of Poland. All data were collected from March 2018 to August 2018. The survey was completed by 439 students: 328 female students (75%) and 111 male students (25%) aged 18 to 50 years (Table 1). All participants completed a battery of online questionnaires: (1) a demographic questionnaire, (2) the Sleep Paralysis Experiences and Phenomenology Questionnaire (SP-EPQ), (3) The PTSD Checklist (PCL), (4) The State-Trait Anxiety Inventory (STAI-T), and (5) The Penn State Worry Questionnaire (PSWQ).

### 2.2. Materials

#### 2.2.1. Demographic Questionnaire

A demographic questionnaire was designed for the current study to collect relevant personal data. The questionnaire assessed two domains: (1) Personal Data included gender, age, height, weight, size of the city in which they live, university profile; (2) lifestyle data included cigarette smoking (the number of cigarettes smoked during the day), the number of hours of sleep during 24 h period (daytime and night-time), alcohol consumption (type of alcohol and frequency of intake during the month), the use of dietary energizers (caffeine and caffeine-containing products), sports (number of hours per week devoted to physical activity).

#### 2.2.2. Sleep Paralysis Experiences and Phenomenology Questionnaire

The Sleep Paralysis Experiences and Phenomenology Questionnaire (SP-EPQ), was designed by Baland Jalal and Devon Hinton to assess the frequency of SP episodes, the presence of psychological and somatic symptoms, the prevalence indices, and the level of knowledge about the experience [23]. The questionnaire has been used in Italy and Turkey [15,17,34]. It is an extended version of the Sleep Paralysis Questionnaire (SPQ) previously used in Cambodia [6], Nigeria [35], China, America [36,37], Egypt, and Denmark [14,23]. To adapt the questionnaire for the Polish student demographic, the questionnaire was translated into Polish by a professional translator, verified by a second professional translator, edited by a native speaker with a medical degree, and subsequently checked by a proof-reader.

The SP-EPQ consists of 17 open and closed questions regarding the frequency of SP episodes (e.g., lifetime, last year and month), the average duration of SP episodes, and emotions felt during the episode. The questionnaire also contains items assessing somatic symptoms accompanying SP, the nature of hallucinations during SP, causal explanations of SP, and measures taken to prevent further episodes of SP [23]. The first question of the questionnaire is formulated as follows: "Some people experienced an event in which they could not move their arms, legs or speak during sleep or waking up, even though they wanted to do it: Have you ever had such an experience?" If participants answered yes to this question, they were asked to describe the episode, thus allowing confirmation that the experience was in fact SP.

#### 2.2.3. The PTSD Checklist (PCL-5)

The PTSD Checklist (PCL-5) measure used in the current study is the latest Polish version of the PCL questionnaire developed for the DSM-5 [38,39,40]. The PCL-5 consists of 20 items pertaining to the severity of PTSD symptoms assessed on a 5-point scale from 0 (not at all) to 4 (very strongly). The Cronbach’s alpha coefficient for this study is 0.92.

#### 2.2.4. The State-Trait Anxiety Inventory (STAI)

In the study, we used the Polish version of STAI of the Psychological Test Laboratory of the Polish Psychological Association [41,42]. We used the fear anxiety subscale as an attribute (X-2 subscale), consisting of 20 items. On a 5-point Likert-scale, the respondents mark to what extent the behaviour described by the statement is typical for them. The measure is scored by summing all the items. The Cronbach’s alpha coefficient for this study is 0.88.

#### 2.2.5. The Penn State Worry Questionnaire (PSWQ)

The PSWQ [43] of Janowski’s adaptation [44] consists of 16 statements describing various manifestations of worry. The respondents indicate on a 5-point Likert-scale to what extent the behaviour described by the statement is typical for them. Responses range from 1 (not at all typical for me) to 5 (very typical for me). The minimum theoretical score is 16 with a maximum of 80 in which higher scores indicate a higher tendency to worry. The Cronbach’s alpha coefficient for this study is 0.94.

### 2.3. Statistical Analysis

The statistical calculations were performed using Statistica (STATISTICA, version 12; StatSoft, Inc, Tulsa, OK, USA). Descriptive statistics were used to describe the group characteristics. We applied a chi-squared test (χ2) to compare demographic factors in the examined groups and a Shapiro–Wilk test to assess the distribution of quantitative variables. To examine the null hypothesis, the Mann–Whitney test was used. We assessed the relationship between not normally distributed continuous and ordinal variables using Spearman’s rank-order correlation test. For analyses, *p* ≤ 0.05 was considered statistically significant.

The project was approved by the Ethics Committee of Medical University of Lublin (the project identification code: KE-0254/125/2017) and performed according to the Declaration of Helsinki guidelines (http://www.nil.org.pl).

## 3. Results

### 3.1. Frequency of Occurrence and Symptomatology of SP

Among the 439 students, 140 individuals (31.89%; 95% CI: 27.5–36.3%) experienced at least one SP episode in their lifetime. The 140 students who experienced SP ((SP+) 31.89%), and the remaining 299 students who did not experience SP ((SP−) 68.11%) did not differ significantly in terms of age or sex. For a comparison of demographic characteristics of SP experiencers and non-SP experiencers, see Table 1.

Among people who suffered from SP, 77% (*n* = 108) have experienced at least one episode in the last year, 34% (*n* = 47) have experienced it 4 times or more in the last year, 29% (*n* = 41) at least one time in the last month. In the SP+ group, 93% (*n* = 130; 95% CI: 87.6–96.7%) reported being afraid during the course of the SP, and 46% (*n* = 64; 95% CI: 36.7–53.3%) felt fear of death.

The majority of respondents, 66% (*n* = 92; 95% CI: 57.8–73.7%), confirmed the occurrence of SP− induced hallucinations involving different sensory modalities (Table 2). Ninety-four percent (*n* = 132) of those who had SP (*n* = 140; 95% CI: 90.4–98.2%), experienced at least one type of somatic symptom (Table 3).

### 3.2. The Relationship between SP and the Severity of Anxiety Symptoms

The severity of anxiety symptoms, the occurrence of PTSD, and a tendency to worry did not differ between the SP sufferers and individuals who had not experienced SP (*p* > 0.05). Among males who experienced SP 4 or more times, the severity of STAI symptoms was higher than among males who experienced SP less than 4 times (*p* < 0.05). Interestingly, this correlation was not found among females experiencing SP; the number of episodes did not correlate with the severity of anxiety symptoms (*p* > 0.05).

### 3.3. The Relationship between Frequency of SP and Lifestyle Variables

There was no correlation between the number of SP episodes experienced and lifestyle variables such as body mass index (BMI), amount of coffee consumed, number of cigarettes smoked, or time spent engaged in physical activity (*p* > 0.05).

However, SP frequency and sleep duration revealed significant gender differences. Among males, the amount of time devoted to sleep during the academic year was positively correlated with the number of lifetime SP episodes (R = 0.34, *p* < 0.05). Among women, a negative correlation was found between the amount of sleep during the academic year and the number of SP episodes in the last year (R = −0.24, *p* < 0.05) and lifetime rates of SP (R = −0.20, *p* < 0.05).

Body position during sleep onset had some effect. Fifty-four percent of subjects experienced SP while sleeping on their backs (*n* = 75). Only 8% experienced it while sleeping on the stomach (*n* = 11), and 38.5% stated that sleep position was not important (*n* = 54). Among females, 51.5% (*n* = 53; 95% CI: 42.6–62.4%) experienced SP while sleeping on the back, 9.7% on the stomach (*n* = 10; 95% CI: 4.0–15.8%); among males 59.5% on the back (*n* = 22; 95% CI: 41.4–76.3%) and on the stomach 2.7% (*n* = 1; 95% CI: −3.0–9.0%). However, these differences were not statistically significant.

Body position during sleep offset was also recorded. Post hoc analyses revealed that those subjects who stated that sleep position was not important experienced more SP episodes compared to subjects who slept on their back. The differences remained significant even after a Bonferroni adjustment (Z = 2.99, *p* = 0.0085).

### 3.4. Factors Affecting the Severity of Somatic and Psychopathological Symptoms in People with SP

Among women, the number of somatic symptoms during SP was positively correlated with the number of cigarettes smoked (R = 0.25, *p* < 0.05) and negatively correlated with time allocated to sleep during the academic year (R = 0.21, *p* < 0.05). Among males, the number of such somatic symptoms experienced during SP was positively correlated with the number of cigarettes smoked (R = 0.37, *p* < 0.05) and the amount of coffee consumed (R = 0.45, *p* < 0.05). However, when looking specifically at hallucinations, there was no relationship between the number of SP-related hallucinations and lifestyle variables (the amount of sleep, coffee drinks consumed, cigarettes smoked).

A positive association was found between somatic symptoms and the severity of PTSD symptoms (R = 0.20, *p* < 0.05). The correlation between lifestyle-related SP risk factors and the number of SP symptoms for all study participants is shown in Table S1. The severity of somatic symptoms was also associated with anxiety symptoms. People that have a fear of death during SP had greater severity of somatic symptoms compared to people who did not fear death (*p* < 0.05). However, the experience of visual hallucinations was not associated with the fear of death or the severity of other anxiety-related symptoms (*p* > 0.05).

## 4. Discussion

Our study is the first of its kind to assess the prevalence of SP in Poland. With this study, we sought to better define SP symptomatology and assess possible causal factors that affect the frequency and severity of SP episodes. We analysed a student population because current research reveals that SP occurs significantly more frequently in student populations as compared to the general population.

We found that the frequency of SP episodes in the student population of Lublin, Poland is 32% and is slightly higher than the average rate of SP prevalence in the world which is estimated at 28.3% [1]. In addition, 34% of the subjects in our study have experienced SP at least four times in the last year, which meets the criteria of recurring isolated sleep paralysis (RISP) [45]. The prevalence of SP episodes for student populations around the world varies to some extent. The highest SP prevalence was recorded in Peru where 55.8% of students experienced at least one episode of SP [46]; in Canada, 29–41.9% of students experienced SP [9,47]; in Japan, 38.9–43% [12,13,14,15,16,17,18,19,20,21,22,23,24,25,26,27,28,29,30,31,32,33,34,35,36,37,38,39,40,41,42,43,44,45,46,47]; in Egypt, 43% [25]; in Nigeria, 26.2–44.2% [48,49]; in Kuwait, 28.8%; in Sudan, 29.9% [50]; and in the USA, 24.5–25% [50,51]. Despite the significant environmental, economic, social, and cultural differences among these countries, the prevalence of SP remains significantly higher in the student population as compared to the general population. In Europe, only one study estimated the prevalence of SP in a group of students was carried out. Previous research reveals that 19.9% of Irish students experienced SP at least once [52]. Our study reveals that 32% of Polish students experienced SP at least once—a figure that is significantly higher. This is surprising given the geographic proximity and cultural similarity shared between Ireland and Poland.

Our study confirmed that SP is associated with feelings of intense fear. Ninety-three percent of study participants reported that they experienced fear during SP, and 46% reported a specific fear of death. The most similar result was obtained in a Canadian study in which 90% of participants reported anxiety [21]. For comparison, in Ireland, fear was reported by 82% and fear of death by 39.8% [52]. In the USA, the Paradis et al. study reported that over 50% of respondents felt fear, and 52% felt fear of death [51]. In the Sharpless and Grom study, 75.64 % felt fear [20]. Many other studies confirm that the SP is accompanied by extreme fear [6,22,53,54,55,56]. 

Our study participants reported both the presence of somatic symptoms and psychological hallucinations. Respondents reported both hypnagogic and hypnopompic hallucinations, i.e., hallucinations before falling asleep and before waking up. The incidence of any hallucinations was 66%. The most common types of hallucinations were visual, 37%. This finding aligns with similar findings reported in Ireland, 53%; in the Czech Republic, 11%; and in Nigeria, 32.6% [48,52,57].

In our study, the reported symptoms were dominated by physical sensations at 92% compared to 66% hallucinations. The most frequently reported somatic symptom was perceived feelings of the heart beating faster (76% of respondents). Other studies have also reported the presence of symptoms such as feelings of pressure [9,21,29,51], difficulty breathing, pain, light-headedness, and dizziness [21], smothering, numbness, vibrating, tingling sensations and feeling numbness [29]. Hinton et al. 2005, described the presence of multiple somatic symptoms such as palpitations and shortness of breath during sleep paralysis among Cambodian refugees that include extremely high rates of panic [6,53]. 

In most studies conducted around the world, the focus has mostly been on hallucinations and has largely omitted the physical symptomatology of SP. The questionnaire we used contained 12 questions about somatic symptoms that may accompany SP. In the commonly used Waterloo Unusual Sleep Experiences Questionnaire VIIIa [58], the feeling of pressure on the chest was classified as a type of hallucination coded as SP− “incubus” [58]. The type “incubus” refers to feelings of pressure on the chest, shortness of breath, or the impression that some creature is sitting on the chest of the person experiencing SP [9]. There is no consensus on the categorization of chest pressure as a type of psychological hallucination or physical symptom associated with stress or impaired breathing during SP [9,47,51,53].

According to Jalal’s “panic-hallucination (PH) model of sleep paralysis,” somatic experiences during SP, such as impaired breathing or chest tightness, may be related to disruptions of the REM sleep phase: hypoxia; hypercapnia; occlusion of the airways; and rapid, shallow breathing [7]. The presence of these symptoms in combination with the paralysis of the body during SP can lead to increased fear and fear for one’s own life. Stress associated with experiencing these negative emotions can trigger the so-called “reaction of fight or flight” of the amygdala and exacerbate feelings of panic [7]. When attempting to move the limbs during an SP episode, in the absence of suppression of proprioceptive feedback, there may be an increase in the sensation of tension and bodily pressure and even pain and limb contraction [59]. Additionally, SP-related somatic symptoms also occur during panic attacks and are included in the ICD-11 criteria for this diagnosis [32]. Thus, these factors combined paint a complicated picture of SP-related hallucinations as perhaps both psychological and somatic. 

Among the surveyed students, 52% of them felt pressure on the chest during SP. In Ireland, this was close to 48% [52]. In studies conducted outside Europe, the frequency of this symptom was lower, e.g., Kuwait, 19.2%; Sudan, 20.7%; Canada, 28.9% [50]; and Japan, 19.7% [47]. Some factors common to Europe, or at least common to Poland and Ireland, may contribute to this, but it is unclear if it may be a cultural factor or due to some other element.

The most important factor we have noticed vis-à-vis the prevalence of SP was the length of sleep. This relationship is shaped quite differently for both sexes. For women, we observed a negative correlation between having SP and the number of hours slept during the academic year, while in men the correlation was positive. Our research is in line with the results of other authors [54,59,60,61], which showed that among Japanese teenagers, the prevalence of SP was higher in those who slept less than 5 h or more than 9 h per 24 h period [60].

The relationship between the occurrence of SP and sleep duration is not clear. According to some researchers, sleep duration and SP are not related [62,63,64]. Due to inconsistent results on this topic, further studies are needed to explore possible predisposing factors. Other research stresses predisposing factors for SP: poor subjective assessment of sleep quality [54,59,60,61,64,65], sleep deprivation [5], symptoms of insomnia [26,66], napping during the day [54,59,60].

Another important factor that affects the course of SP is the position of the body. Our research confirms that the position of the body when lying on one’s back promotes the occurrence of SP [67,68]. In a study carried out by Spanos et al. (1995), as many as 70% of students reported having experienced SP when lying on their back [69]. The results of Fukuda et al. (1998) showed that 58% of Canadian and 84% of Japanese students experienced SP in a position on the back, while in the entire study population 4% of Canadians and 41% of Japanese declared that they usually sleep on their backs [47].

The relationship between SP-related anxiety symptoms and post-traumatic stress symptoms demonstrated in previous studies has not been confirmed in our study. In the majority of cases studying SP and anxiety or post-traumatic stress symptoms, the research was focused on psychiatric patients, most of whom lived in countries with significant cultural differences from Poland [6,53,55]. The strong relationship between PTSD and anxiety symptoms was shown among Cambodian refugees who were treated in psychiatric clinics and among patients of psychiatric hospitals in China and in the USA [6,53,55]. In Cambodia, among psychiatric patients, the prevalence of SP is high, with 49% having experienced SP in the last year, with the occurrence of the phenomenon almost always attributed to the action of supernatural beings such as ghosts and demons [6,14,53]. Here, the significant cultural differences may come into play as in Poland, and SP is mostly interpreted as a condition caused by stress, fatigue, poor sleep hygiene, and other lifestyle factors—not as a cultural phenomenon.

In studies conducted by Jalal and colleagues among Egyptian students experiencing SP, the severity of PTSD symptoms, anxiety symptoms, and the tendency to worry was higher compared to peers who do not experience it [25]. Among the participants of the study, 11% explained the phenomenon of SP as a genie attack. In the general population of this country, as much as 41% interpreted the phenomenon as the effect of the genie [15].

In our study, men who experienced SP four or more times had a higher severity of anxiety symptoms on the STAI scale, compared to men who experienced SP less than 4 times (*p* < 0.05). This suggests that the high levels of anxiety as a trait may be a risk factor for the occurrence of SP episodes among men. The greater severity of anxiety symptoms was associated with a greater number of somatic symptoms present during SP. Additionally, a positive relationship between somatic symptoms and the severity of symptoms of PTSD measured by the PLC-5 scale was also demonstrated among participants. The results obtained by us suggest a relationship between anxiety symptoms and PTSD symptoms and the course and occurrence of SP.

The course of SP and the severity of somatic symptoms may be affected by cigarette smoking and caffeinated beverage consumption. Cigarette smoking is associated with a higher incidence of SP [60]. However, other studies do not confirm this relationship [54,64], and the influence of caffeine on the frequency of SP was ruled out by current studies [61]. Furthermore, our research sheds new light on this problem. Until now, the influence of these psychoactive substances on the severity of SP symptoms has not yet been studied. Our finding seems to be worth considering due to the fact that nicotine [70,71,72] and caffeine [73] have a negative effect on the quality of sleep.

Other such lifestyle variables should be studied in the future, especially in countries where SP is interpreted as caused by lifestyle issues rather than supernatural forces. Additionally, studies involving genetic, neurophysiological, and neuropsychological analysis in different populations could be helpful in further distinguishing among possible factors.

Limitations of this study include the sample being limited to university students from Lublin province and reliance on online self-report measures alone. It should be taken into account that SP sufferers were more willing to respond to the survey than people who did not experience this phenomenon. This could have an impact on the prevalence of SP among Polish students. The study had a limited selection of causative factors. We assume that there are other risk factors affecting the occurrence of SP in the Polish student population. In the future, it would be reasonable to take into account other potential causal factors be it behavioural (e.g., naps during day, medicines), psychological (e.g., stress coping capacity, sleep quality) or chronic disorder symptoms (e.g., narcolepsy, depression). Further research is necessary to determine the risk factors for SP in the Polish population.

The results of our study indicate that a significant proportion of students experience recurrent isolated sleep paralysis and that this phenomenon is associated with great fear and physical discomfort, which may further exacerbate such anxiety. Frequent and recurrent SP episodes can significantly reduce the quality of life of people experiencing them and can also negatively impact students’ lives. The scale of the phenomenon revealed in the study requires a deeper analysis. In addition, it seems that scientific awareness about this disorder is not widely spread in Poland. There are no evidence-based (i.e., empirically supported) available forms of therapy for people experiencing SP. Therefore, further research on SP including determining the effectiveness of available forms of therapy should be encouraged (e.g., Jalal, 2016, 2017) [7,74].

## 5. Conclusions

1. The incidence of SP in the Lublin, Poland, student population was 32% and was slightly higher than the average prevalence in the world, which is estimated at 28.3%.

2. The clinical picture of SP was dominated by somatic symptoms, which were reported by 94% of respondents (most often tachycardia, 76%), 93% fear (most often fear of death, 46%), and 66% hallucinations (most often visual hallucinations, 37%).

3. The number of episodes of SP was related to the length of sleep and the position of the body during its course and, in the absence of correlation with BMI, the amount of coffee consumed, the number of cigarettes smoked, and the time spent on physical activity.

4. The severity of somatic symptoms during SP correlated with lifestyle variables such as sleep duration, smoking habits, and coffee consumption as well as with increased anxiety-related symptoms.

## Figures and Tables

**Table 1:** Participants’ demographic characteristics.

Sample Type	*N*	% Female (*n*)	% Male (*n*)	Age	
				*M* (*SD*)	RNG
SP +	140	31.4 (103)	33.3 (37)	22 (3.92)	18–50
No SP	299	68.6 (225)	76.7 (74)	22 (4.36)	18–50

Note. SP+ = participants with at least one lifetime episode of SP; No SP = individuals who have not experienced SP.

**Table 2:** The frequency of occurrence of hallucinations during sleep paralysis (SP) in the studied group of students.

Type of Hallucinations	Visual		Auditory		Tactile		Olfactory	
	N	% (95% CI)	N	% (95% CI)	N	% (95% CI)	N	% (95% CI)
Group SP+	52	37 (29.0–45.3)	44	31 (23.6–39.2)	35	25 (17.7–32.3)	3	2 (−0.29–4.6)

Note. SP+ = participants with at least one lifetime episode of SP; N = number of responders; 95% CI = 95% confidence interval.

**Table 3:** The frequency of occurrence of somatic symptoms during SP in the studied group of students.

Sample Type	Group SP+		Females SP+		Males SP+		Differences Between Females and Males
	N	% (95% CI)	N	%	N	%	
At least one symptom	132	94 (57.8–73.7)	97	94	35	95	*p > 0.05*
Pressure on chest	72	51 (43.0–59.8)	53	51	19	51	
Unable to breathe	51	35 (28.4–44.5)	41	40	10	27	
Chest pain/discomfort	54	39 (30.4–46.7)	41	40	13	35	
Feeling of chocking	18	13 (7.2–18.5)	16	16	2	5	
Nausea, abdominal distress	7	5 (1.4–8.7)	5	5	2	5	
Feeling dizzy, unsteady	44	31 (23.6–39.2)	34	33	10	27	
Sweating	71	51 (42.3–59.1)	53	51	18	49	
Trembling/shaking	51	36 (28.4–44.5)	36	35	15	41	
Heart palpitations	106	76 (68.5–82.9)	80	78	26	70	
Chills or hot flushes	48	34 (26.3–42.2)	38	37	10	27	

Note. SP+ = participants with at least one lifetime episode of SP; N = number of responders; 95% CI = 95% confidence interval; *p* = *p*-value.

## Supplementary Materials

The following are available online at https://www.mdpi.com/1660-4601/17/10/3529/s1, Table S1: Correlation between lifestyle related SP risk factors and the number of SP symptoms.
