# Supplementary material for: Prevalence and Clinical Picture of Sleep Paralysis in a Polish Student Sample
Source: Int J Environ Res Public Health. 2020 May 18;17(10):3529. doi: 10.3390/ijerph17103529 (PMC7277803; doi:10.3390/ijerph17103529)
Supplement: Supplementary file 1 [file ijerph-17-03529-s001.pdf]

Table S1. Correlation between lifestyle related SP risk factors and the number of SP symptoms.

	**Cups of Coffee Drank**	**Number of Cigerettes Smoked**	**Hours of Sleep During the Academic Year**	**Number of SP Episodes in the Last Year**	**Number of SP Episodes in One's Lifetime**
Number of somatic symptoms	0.20	0.27	-0.24	0.21	0.22
Number of psychopathological symptoms	-	-	-	-	-
Symptoms combined	-	0.26	-0.25	0.23	0.23

The correlation between lifestyle related factors and the number of SP symptoms is presented using the Spearman's rank correlation coefficient (rs). Significance coefficients, $p < 0.05$.
